# Data article on genes that share similar expression patterns with EEF1 complex proteins in hepatocellular carcinoma

**DOI:** 10.1016/j.dib.2020.105162

**Published:** 2020-01-23

**Authors:** Burcu Biterge Süt

**Affiliations:** Niğde Ömer Halisdemir University, Faculty of Medicine, Department of Medical Biology, Turkey

**Keywords:** EEF1 complex, Cancer, Gene expression, Data mining

## Abstract

Eukaryotic Elongation Factor complex 1 (EEF1) consists of six subunits namely EEF1A1, EEF1A2, EEF1B2, EEF1D, EEF1E1 and EEF1G. Recently we showed that EEF1 complex proteins might play critical roles in cancer [1]. This article provides data on genes that share similar expression patterns with EEF1 complex proteins in cancer by analyzing RNA expression data using GEPIA online tool. Correlation analysis was performed on selected genes in a pairwise manner and the Pearson coefficients were automatically calculated by the GEPIA online tool. These data can be useful for future studies directed towards understanding the mechanisms by which EEF1 complex proteins affect in cancer pathogenesis.

**Table:** Specifications Table

Subject	Cancer research
Specific subject area	Protein expression in cancer
Type of data	Raw Table Figure
How data were acquired	GEPIA online tool
Data format	Raw and analyzed
Parameters for data collection	RNA expression levels
Description of data collection	By analyzing RNA expression data using online tools
Data source location	The data published here are based upon data generated by the TCGA Research Network (https://www.cancer.gov/tcga).
Data accessibility	The raw data files are provided by the TCGA Research Network and can be reached at https://xenabrowser.net/datapages/?dataset=TCGA.LIHC.sampleMap%2FHiSeqV2&host=https%3A%2F%2Ftcga.xenahubs.net&removeHub=https%3A%2F%2Fxena.treehouse.gi.ucsc.edu%3A443. Analyzed data are shared with this article.
Related research article	Biterge-Sut B. 2019. Alterations in Eukaryotic Elongation Factor complex proteins (EEF1s) in cancer and their implications in epigenetic regulation. Life Sci. 238:116977. https://doi.org/10.1016/j.lfs.2019.116977

**Table:** 

**Value of the Data** • Cancer pathogenesis relies largely on aberrant protein expression. • EEF1 complex proteins, together with other genes that share similar expression patterns with them, could affect carcinogenesis [1]. • These data can be useful for future studies directed towards understanding the mechanisms by which EEF1 complex proteins affect in cancer.

## 1 Data description

**Fig. 1:**
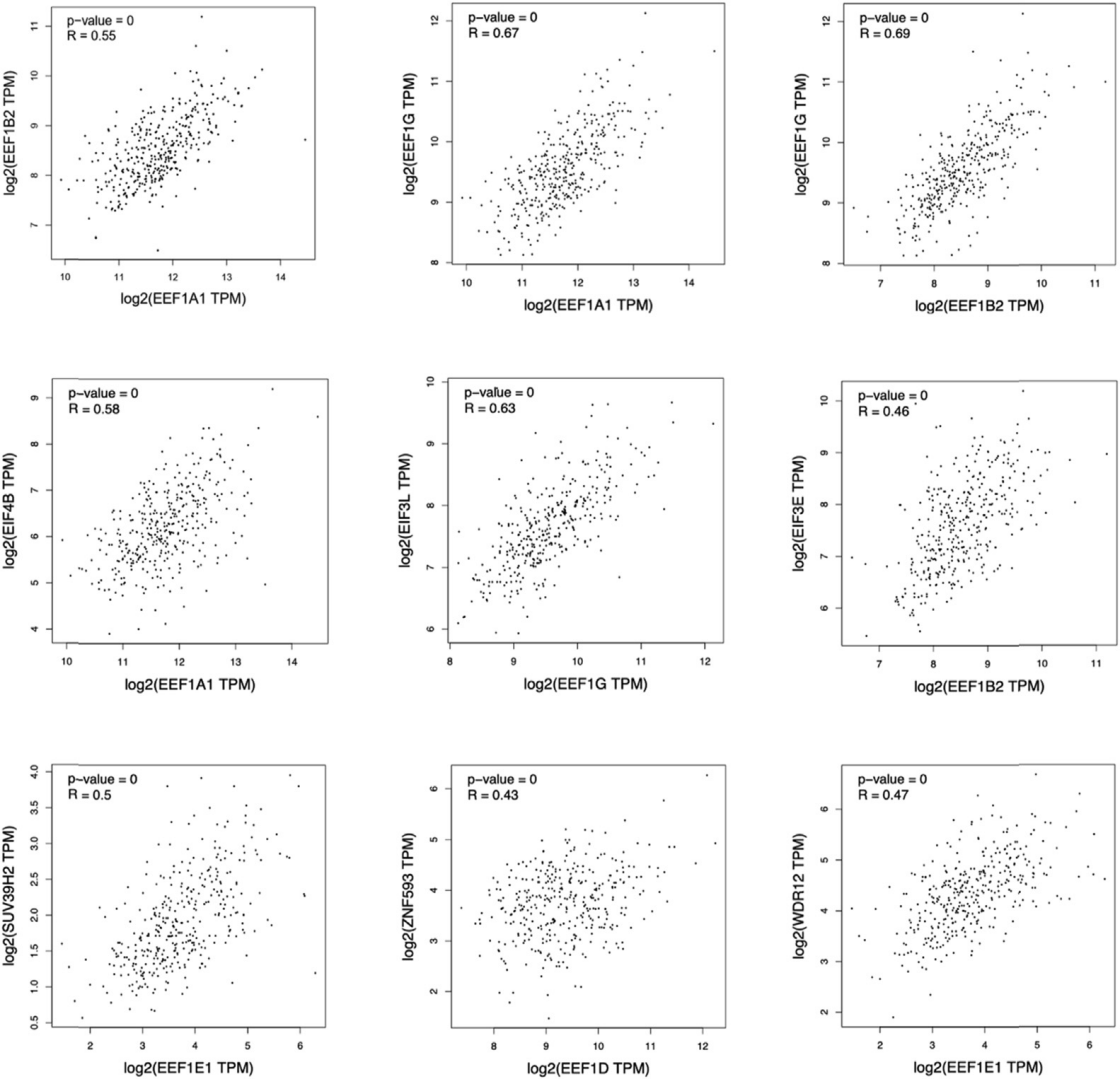
Analysis of gene expression correlation between selected gene pairs using datasets.

The datasets that were analyzed in this data article were acquired by the GEPIA tool from the data generated by The Cancer Genome Atlas (TCGA) Research Network (dataset ID: TCGA.LIHC.sampleMap/HiSeqV2) [2,3]. For each EEF1 complex protein, a list of genes with similar expression to the input gene were created and ranked according to their Pearson coefficients. The first 50 genes were selected for each EEF1 protein (Table 1). Analysis of gene expression correlation between selected gene pairs is given in Fig. 1.

**Table 1:** Genes that share similar expression patterns with EEF1 complex proteins in cancer ranked by Pearson correlation coefficient (PCC).

EEF1A1			EEF1A2			EEF1B2		
Gene symbol	Gene ID	PCC	Gene symbol	Gene ID	PCC	Gene symbol	Gene ID	PCC
EEF1A1P5	ENSG00000196205.8	0.72	CDK5R2	ENSG00000171450.5	0.74	RPS3A	ENSG00000145425.9	0.68
RPL5	ENSG00000122406.12	0.72	SYP	ENSG00000102003.10	0.74	RPS13	ENSG00000110700.6	0.66
EEF1A1P6	ENSG00000233476.3	0.69	ATP1A3	ENSG00000105409.15	0.69	RPS23	ENSG00000186468.12	0.66
EEF1A1P9	ENSG00000249264.1	0.68	FAIM2	ENSG00000135472.8	0.69	RPS18	ENSG00000231500.6	0.66
TPT1	ENSG00000133112.16	0.68	SYT5	ENSG00000129990.14	0.68	RPS27A	ENSG00000143947.12	0.65
EIF4B	ENSG00000063046.17	0.68	CPLX2	ENSG00000145920.14	0.68	RPL10A	ENSG00000198755.10	0.65
RPL3	ENSG00000100316.15	0.68	TMEM179	ENSG00000258986.6	0.68	RPS6	ENSG00000137154.12	0.64
RPL10A	ENSG00000198755.10	0.68	FAM163B	ENSG00000196990.8	0.68	RPLP0	ENSG00000089157.15	0.64
RPS3A	ENSG00000145425.9	0.68	RUNDC3A	ENSG00000108309.12	0.67	RPS15A	ENSG00000134419.15	0.64
IGBP1	ENSG00000089289.15	0.68	DUSP26	ENSG00000133878.8	0.67	RPS7	ENSG00000171863.12	0.63
EEF1G	ENSG00000254772.9	0.66	STMN2	ENSG00000104435.13	0.67	RPL5	ENSG00000122406.12	0.63
EEF1A1P12	ENSG00000214199.3	0.66	TAGLN3	ENSG00000144834.12	0.66	EEF1B2P3	ENSG00000232472.1	0.63
RPL31	ENSG00000071082.10	0.65	GS1-72M22.1	ENSG00000272163.1	0.66	RPL26	ENSG00000161970.12	0.62
RPS23	ENSG00000186468.12	0.65	UNC5A	ENSG00000113763.10	0.66	RPL31	ENSG00000071082.10	0.61
RPS4X	ENSG00000198034.10	0.65	PTPRN	ENSG00000054356.13	0.66	RPS8	ENSG00000142937.11	0.61
RPL26	ENSG00000161970.12	0.64	APLP1	ENSG00000105290.11	0.65	RPL27	ENSG00000131469.12	0.61
RPS13	ENSG00000110700.6	0.63	CELF4	ENSG00000101489.18	0.65	RPL36A	ENSG00000241343.9	0.6
NACA	ENSG00000196531.10	0.63	INA	ENSG00000148798.9	0.65	EEF1G	ENSG00000254772.9	0.6
LRRC75A-AS1	ENSG00000175061.17	0.63	CCDC184	ENSG00000177875.4	0.65	RPS14	ENSG00000164587.11	0.6
NPM1	ENSG00000181163.13	0.63	TMEM130	ENSG00000166448.14	0.65	RPS29	ENSG00000213741.8	0.6
RPL4	ENSG00000174444.14	0.62	HCN1	ENSG00000164588.4	0.64	RPSA	ENSG00000168028.13	0.59
RPL34	ENSG00000109475.16	0.62	RIMBP2	ENSG00000060709.13	0.64	RPL39	ENSG00000198918.7	0.59
HNRNPA1	ENSG00000135486.17	0.61	SPTBN4	ENSG00000160460.15	0.64	RPL21	ENSG00000122026.10	0.59
RPS25	ENSG00000118181.10	0.61	BEX1	ENSG00000133169.5	0.64	RP11-572P18.1	ENSG00000220842.6	0.59
EIF3L	ENSG00000100129.17	0.61	CAMK2B	ENSG00000058404.19	0.64	RPL7A	ENSG00000148303.16	0.59
GNB2L1	ENSG00000204628.11	0.6	SYT4	ENSG00000132872.11	0.64	RPS12	ENSG00000112306.7	0.57
EIF4BP7	ENSG00000225031.1	0.6	ST8SIA3	ENSG00000177511.5	0.63	RPS10	ENSG00000124614.13	0.57
RPL7A	ENSG00000148303.16	0.6	PDZD7	ENSG00000186862.17	0.63	RPL4	ENSG00000174444.14	0.57
RPS15A	ENSG00000134419.15	0.6	CHRNB2	ENSG00000160716.4	0.63	RPS5	ENSG00000083845.8	0.56
BTF3	ENSG00000145741.15	0.6	SEZ6L2	ENSG00000174938.14	0.63	EEF1A1	ENSG00000156508.17	0.56
NSA2	ENSG00000164346.9	0.6	GPR22	ENSG00000172209.5	0.63	RPS3	ENSG00000149273.14	0.56
RSL24D1	ENSG00000137876.9	0.6	NAP1L5	ENSG00000177432.6	0.63	RPL37A	ENSG00000197756.9	0.55
EEF1A1P19	ENSG00000249855.1	0.6	MAPK8IP2	ENSG00000008735.13	0.62	RPS17	ENSG00000182774.10	0.55
RPS27A	ENSG00000143947.12	0.59	KIF1A	ENSG00000130294.14	0.62	RPS24	ENSG00000138326.18	0.55
RPS14	ENSG00000164587.11	0.59	RALYL	ENSG00000184672.11	0.62	RPS11	ENSG00000142534.6	0.55
RPL41	ENSG00000279483.1	0.59	BEGAIN	ENSG00000183092.15	0.62	RPL14	ENSG00000188846.13	0.55
RPL15	ENSG00000174748.18	0.58	DYNC1I1	ENSG00000158560.14	0.62	RPL7	ENSG00000147604.13	0.55
CCNI	ENSG00000118816.9	0.58	SYNGR3	ENSG00000127561.14	0.62	RPS4X	ENSG00000198034.10	0.55
RPS6	ENSG00000137154.12	0.58	CALY	ENSG00000130643.8	0.62	EIF3E	ENSG00000104408.9	0.55
RPL10	ENSG00000147403.16	0.58	JPH4	ENSG00000092051.16	0.61	RPL17	ENSG00000265681.6	0.55
RPL11	ENSG00000142676.12	0.57	SCAMP5	ENSG00000198794.11	0.61	RPL10	ENSG00000147403.16	0.54
RPL14	ENSG00000188846.13	0.57	UCHL1	ENSG00000154277.12	0.61	RPS9	ENSG00000170889.13	0.54
RPL17	ENSG00000265681.6	0.57	RAB3C	ENSG00000152932.7	0.61	BTF3	ENSG00000145741.15	0.54
RPS8	ENSG00000142937.11	0.57	SCN3B	ENSG00000166257.8	0.61	RPL29	ENSG00000162244.10	0.54
RPL12	ENSG00000197958.12	0.57	FBLL1	ENSG00000188573.7	0.6	RPL34	ENSG00000109475.16	0.54
EEF1B2	ENSG00000114942.13	0.56	TERF2IP	ENSG00000166848.5	0.6	RPL12	ENSG00000197958.12	0.54
RPL22	ENSG00000116251.9	0.56	SYT1	ENSG00000067715.13	0.6	RPS21	ENSG00000171858.17	0.54
RPL24	ENSG00000114391.12	0.56	SLC8A2	ENSG00000118160.13	0.6	RPL27A	ENSG00000166441.12	0.54
EIF3E	ENSG00000104408.9	0.56	RIPPLY2	ENSG00000203877.7	0.6	RPL11	ENSG00000142676.12	0.53
EEF1A1P7	ENSG00000268222.1	0.56	MAP6	ENSG00000171533.11	0.6	HNRNPA1	ENSG00000135486.17	0.53

EEF1D			EEF1E1			EEF1G		
Gene symbol	Gene ID	PCC	Gene symbol	Gene ID	PCC	Gene symbol	Gene ID	PCC
RPL8	ENSG00000161016.15	0.77	BUB1	ENSG00000169679.14	0.63	RPL3	ENSG00000100316.15	0.76
RPL36	ENSG00000130255.12	0.65	NOL7	ENSG00000225921.6	0.62	RPL7A	ENSG00000148303.16	0.75
RPS9	ENSG00000170889.13	0.64	TFAM	ENSG00000108064.10	0.62	RPL10A	ENSG00000198755.10	0.75
RPLP2	ENSG00000177600.8	0.64	DNAH14	ENSG00000185842.14	0.62	RPS13	ENSG00000110700.6	0.73
RPL30	ENSG00000156482.10	0.61	WDR12	ENSG00000138442.9	0.61	RPLP0	ENSG00000089157.15	0.72
RPL13A	ENSG00000142541.16	0.61	KIF2C	ENSG00000142945.12	0.61	RPS18	ENSG00000231500.6	0.72
FAU	ENSG00000149806.10	0.6	DBF4	ENSG00000006634.7	0.6	RPL13A	ENSG00000142541.16	0.71
RPL27A	ENSG00000166441.12	0.6	MRPL42	ENSG00000198015.12	0.6	RPS9	ENSG00000170889.13	0.71
VPS28	ENSG00000160948.13	0.6	CDC45	ENSG00000093009.9	0.59	RPL31	ENSG00000071082.10	0.71
RPL35	ENSG00000136942.14	0.59	AUNIP	ENSG00000127423.10	0.59	RPL4	ENSG00000174444.14	0.7
RPS19	ENSG00000105372.6	0.58	GART	ENSG00000159131.16	0.59	RPL12	ENSG00000197958.12	0.7
RPL18	ENSG00000063177.12	0.58	CDC25C	ENSG00000158402.18	0.59	RPL11	ENSG00000142676.12	0.7
EDF1	ENSG00000107223.12	0.57	MTFR2	ENSG00000146410.11	0.59	RPS11	ENSG00000142534.6	0.69
RPL37A	ENSG00000197756.9	0.56	TIPIN	ENSG00000075131.9	0.59	RPL7	ENSG00000147604.13	0.69
RPL13	ENSG00000167526.13	0.56	CCT8	ENSG00000156261.12	0.59	RPS8	ENSG00000142937.11	0.68
EXOSC4	ENSG00000178896.6	0.56	MTX2	ENSG00000128654.13	0.59	RPL29	ENSG00000162244.10	0.68
RPS11	ENSG00000142534.6	0.55	TIMM23	ENSG00000265354.3	0.58	RPS27A	ENSG00000143947.12	0.68
RPS28P7	ENSG00000227097.5	0.55	RANBP1	ENSG00000099901.16	0.58	RPS23	ENSG00000186468.12	0.68
UQCC3	ENSG00000204922.4	0.55	VARS	ENSG00000204394.12	0.58	RPL5	ENSG00000122406.12	0.68
RPLP1	ENSG00000137818.11	0.54	SSB	ENSG00000138385.15	0.58	RPL41	ENSG00000279483.1	0.67
RPS2	ENSG00000140988.15	0.54	TPRKB	ENSG00000144034.14	0.58	RPS15A	ENSG00000134419.15	0.67
SART1	ENSG00000175467.14	0.53	THUMPD3	ENSG00000134077.15	0.58	RPS25	ENSG00000118181.10	0.67
RPL12	ENSG00000197958.12	0.53	SASS6	ENSG00000156876.9	0.57	RPS2	ENSG00000140988.15	0.67
ARL6IP4	ENSG00000182196.13	0.53	ORC6	ENSG00000091651.8	0.57	RPL36A	ENSG00000241343.9	0.67
MZT2B	ENSG00000152082.13	0.53	MIS18A	ENSG00000159055.3	0.57	RPL27A	ENSG00000166441.12	0.66
RFXANK	ENSG00000064490.13	0.52	TAF9	ENSG00000273841.4	0.57	RPL10	ENSG00000147403.16	0.66
RPL24	ENSG00000114391.12	0.52	CPSF3	ENSG00000119203.13	0.57	EEF1A1	ENSG00000156508.17	0.66
RPS8	ENSG00000142937.11	0.52	MRPL39	ENSG00000154719.13	0.57	RPL37A	ENSG00000197756.9	0.66
RPS15	ENSG00000115268.9	0.52	EXOSC9	ENSG00000123737.12	0.57	GNB2L1	ENSG00000204628.11	0.66
UBA52	ENSG00000221983.7	0.52	POLR2D	ENSG00000144231.10	0.57	RPS6	ENSG00000137154.12	0.66
RP11-466H18.1	ENSG00000244398.1	0.52	NCAPG	ENSG00000109805.9	0.57	RPL14	ENSG00000188846.13	0.65
RPS21	ENSG00000171858.17	0.52	CENPH	ENSG00000153044.9	0.57	RPS4X	ENSG00000198034.10	0.65
C19orf24	ENSG00000228300.13	0.52	PBK	ENSG00000168078.9	0.56	RPL26	ENSG00000161970.12	0.65
MRPL23	ENSG00000214026.10	0.51	SUV39H2	ENSG00000152455.15	0.56	RPS12	ENSG00000112306.7	0.65
RPL28	ENSG00000108107.12	0.51	UBE2N	ENSG00000177889.9	0.56	NACA	ENSG00000196531.10	0.65
RP11-641D5.1	ENSG00000213178.3	0.51	HMMR	ENSG00000072571.19	0.56	RPS24	ENSG00000138326.18	0.65
GPAA1	ENSG00000197858.10	0.51	FANCD2	ENSG00000144554.10	0.56	RPS3A	ENSG00000145425.9	0.64
RPL36A	ENSG00000241343.9	0.51	ELAVL1	ENSG00000066044.13	0.56	RPS10	ENSG00000124614.13	0.64
RPL32	ENSG00000144713.12	0.51	SPDL1	ENSG00000040275.16	0.56	RPL18	ENSG00000063177.12	0.64
RPL7	ENSG00000147604.13	0.51	MAD2L1	ENSG00000164109.13	0.56	RPL32	ENSG00000144713.12	0.63
MAF1	ENSG00000179632.9	0.51	NUP35	ENSG00000163002.12	0.56	RPS3	ENSG00000149273.14	0.63
RPS20	ENSG00000008988.9	0.51	SGOL1	ENSG00000129810.14	0.56	RPS14	ENSG00000164587.11	0.63
IMPDH2	ENSG00000178035.11	0.5	PSMG1	ENSG00000183527.11	0.56	LRRC75A-AS1	ENSG00000175061.17	0.63
GADD45GIP1	ENSG00000179271.2	0.5	PLK4	ENSG00000142731.10	0.56	RPL23A	ENSG00000198242.13	0.63
RPL38	ENSG00000172809.12	0.5	FAM72A	ENSG00000196550.10	0.56	EIF3L	ENSG00000100129.17	0.62
RPL37	ENSG00000145592.13	0.5	PPM1G	ENSG00000115241.10	0.55	IMPDH2	ENSG00000178035.11	0.62
GSDMD	ENSG00000104518.10	0.5	LRIF1	ENSG00000121931.15	0.55	RPS17	ENSG00000182774.10	0.62
AP003068.9	ENSG00000254501.1	0.5	ZC3H15	ENSG00000065548.17	0.55	RPL39	ENSG00000198918.7	0.62
RPS14	ENSG00000164587.11	0.5	NUP37	ENSG00000075188.8	0.55	RPL22	ENSG00000116251.9	0.62
ZNF593	ENSG00000142684.7	0.49	KIAA1143	ENSG00000163807.5	0.55	RPL36	ENSG00000130255.12	0.62

## 2 Experimental design, materials, and methods

### 2.1 Identification of similarly expressed genes

In order to identify genes that share similar expression patterns with EEF1 complex proteins, GEPIA analysis was performed. GEPIA is an online data repository that uses RNA-Seq datasets based on the UCSC Xena project and allows protein expression analysis from genome-wide DNA and RNA sequencing data (http://gepia.cancer-pku.cn/index.html) [4,5]. First, EEF1A1, EEF1A2, EEF1B2, EEF1D, EEF1E1 or EEF1G were selected as the gene of interest under *Similar Genes* section. Then, the number of genes was set to 50 and the cancer type was set as TCGA Tumor-liver hepatocellular carcinoma. Pearson correlation coefficients (PCCs) were automatically calculated by the GEPIA online tool.

### 2.2 Correlation analysis

The similarity in expression patterns was further evaluated and validated by the *Correlation Analysis* feature of GEPIA tool. For this purpose, genes of interest were set as gene A and gene B. The tool offers statistical analysis based on methods including Pearson, Spearman and Kendall; and uses non-log scale for calculation and uses the log-scale axis for visualization. Among these, Pearson correlation was selected and further confirmed by analyzing the Spearman correlations. The cancer type was set as liver hepatocellular carcinoma.
