# Supplementary material for: Data article on genes that share similar expression patterns with EEF1 complex proteins in hepatocellular carcinoma
Source: Data Brief. 2020 Jan 23;29:105162. doi: 10.1016/j.dib.2020.105162 (PMC6997806; doi:10.1016/j.dib.2020.105162)
Supplement: Multimedia component 2 [file mmc2.docx]

**Additional figure for review but not for publication:**

The correlation between the expression levels of the gene pairs mentioned in Figure 1 has been tested using additional statistical methods namely by calculating the Spearman correlations (below), which showed similar results.

**
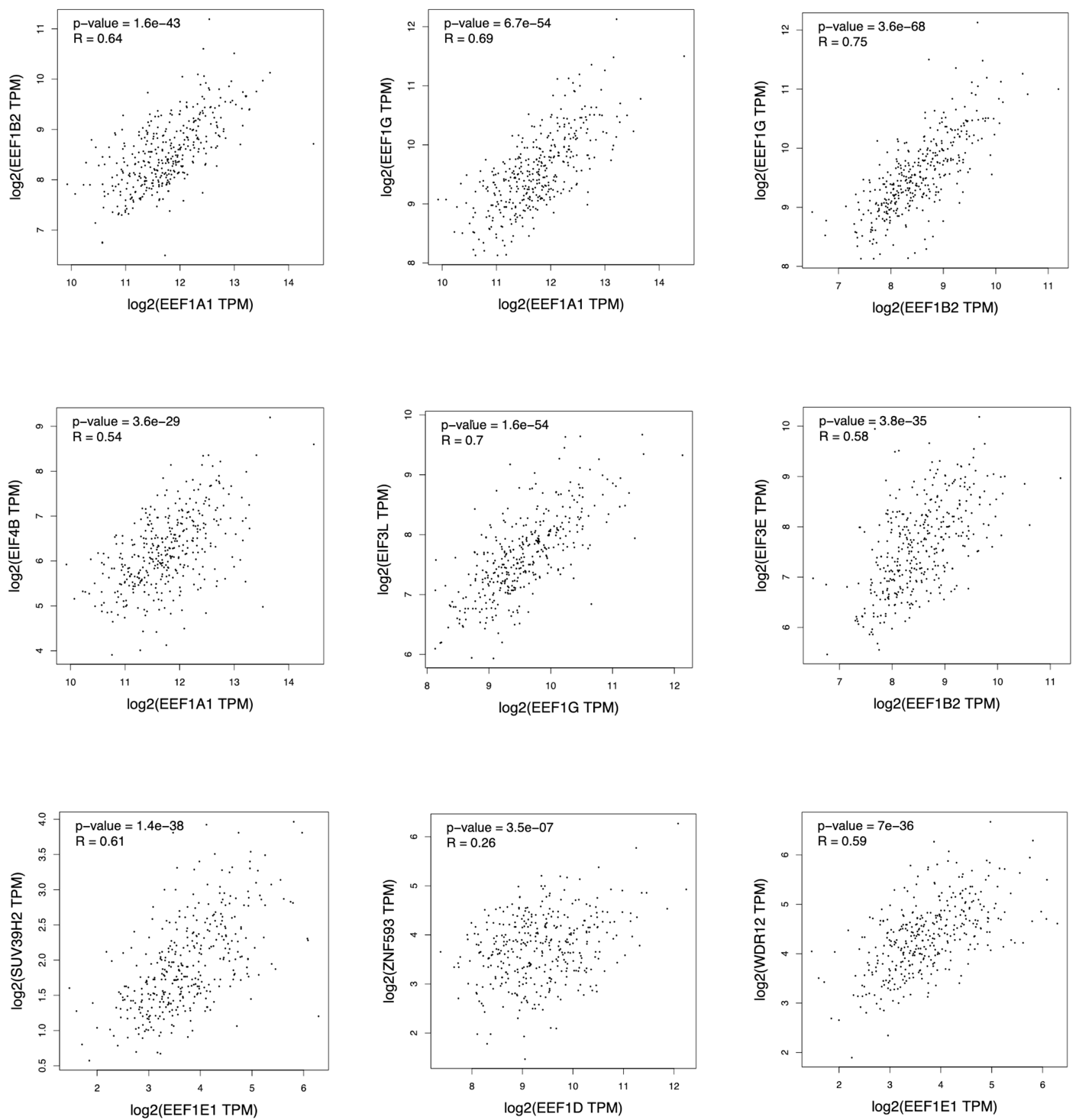
**
